# Poverty-reduction interventions combined with psychological interventions: A systematic literature review

**DOI:** 10.1038/s41598-025-24736-8

**Published:** 2025-11-19

**Authors:** Mimi Tanski, Dannuo Wei, Sangeeta Singh, Mauricio Avendano Pabon, Vikram Kisansingh Bahure, Mark J.D. Jordans, Crick Lund, Sanchari Roy, Rakesh Singh, Atuleisha Thapa, Wietse Anton Tol, Sara Evans-Lacko

**Affiliations:** 1 Department of Psychology, University of Pennsylvania, Stephen A. Levin Building, 425 S. University Ave, Philadelphia, PA 19104, USA; 2 Department of Social Work and Social Administration, University of Hong Kong, Pok Fu Lam, Hong Kong; 3 London School of Economics, Care Policy and Evaluation Centre, Houghton St, London WC2A 2AZ, UK; 4 Department of Epidemiology and Health Systems, Center for Primary Care and Public Health, University of Lausanne, Route de Berne 113, Unisanté, Lausanne 1010, Switzerland; 5 Public First, 20 Victoria St, London SW1H 0NB, UK; 6 Centre for Global Mental Health, Health Service and Population Research Department, Institute of Psychiatry, Psychology and Neuroscience, King’s College London, Strand, London WC2R 2LS, UK; 7 Department of Psychiatry and Mental Health, Alan J Flisher Centre for Public Mental Health, University of Cape Town, Neuroscience Institute, Groote Schuur Hospital Observatory, Anzio Road, 1 st floor, Cape Town, South Africa; 8 Department of Economics, University of Exeter, Rennes Dr, Exeter EX4 4PU, UK; 9 Research Department, Transcultural Psychosocial Organization Nepal (TPO Nepal), Anek Marga, Kathmandu 44600, Nepal; 10 Transcultural Psychosocial Organization Nepal (TPO Nepal), Anek Marga, Kathmandu 44600, Nepal; 11 Section of Global Health, Department of Public Health, University of Copenhagen, CSS Campus, øster Farimagsgade 5, building 9, Copenhagen K DK-1353, Denmark; 12 Athena Research Institute, Faculty of Science, Vrije Universiteit, VU Amsterdam, Main Building (HG-0E), ground floor, wing E, De Boelelaan 1105, Amsterdam 1081 HV, The Netherlands; 13 Department of Mental Health, Bloomberg School of Public Health, Johns Hopkins University, 615 N. Wolfe Street, Baltimore, MD 21205, USA; 14 London School of Economics and Political Science, Care Policy and Evaluation Centre, Houghton St, London WC2A 2AZ, UK; 15 Department of International Development, King’s College London, Strand, WC2R 2LS London, UK

## Abstract

Research increasingly indicates that poverty and mental health are causally and bidirectionally related, creating a vicious cycle of disadvantage. We conducted a systematic review with a quantitative summary of effect sizes synthesizing evidence of interventions combining mental health and poverty-reduction components. Seventeen studies were included, spanning diverse populations and contexts. The extracted outcomes were analyzed by outcome type, follow-up duration and comparator in the narrative analysis and forest plots displayed effect estimates by outcome. The most common psychological components were psychosocial interventions delivered by non-specialists, while poverty-reduction components most often involved cash or asset transfers. Combined interventions compared to inactive controls were more consistently associated with improvements in mental health problems, psychological wellbeing and socioeconomic outcomes. Combined approaches demonstrated relatively consistent benefits when compared to psychological-only interventions but showed more mixed results when compared to poverty-reduction components alone, suggesting that the marginal benefit of adding psychological components may be limited and require attention to contextual and implementation factors. Findings highlight the potential of integrated strategies to address both social and psychological determinants of mental health.

Poverty and mental health problems are major global challenges with significant social and economic impacts. Mental disorders are responsible for one-sixth of years lived with disability (YLDs) making them the leading cause of YLDs. ^1^ Poverty can increase the risk of mental illness^2,3^ and reduce access to quality care and social support. ^4^ Evidence increasingly indicates that poverty and mental health problems are interrelated and mutually reinforcing, creating a vicious cycle of disadvantage and distress^3^ that contributes to persistent socioeconomic inequality and lack of social mobility.

Several systematic reviews have examined the effects of poverty-reduction interventions on mental health and well-being outcomes^5–9^and explored mechanisms through which these interventions could impact on mental health^10^. The reviews suggest there may be a positive, albeit modest, relationship between cash transfer receipt and improved mental health. Another recent review found positive economic impacts of mental health interventions in low- and middle-income countries (LMICs)^11^. Findings from these reviews support the potential for interdisciplinary, multi-sectoral approaches to improve both mental health and socioeconomic outcomes.

Yet both mental health and poverty-reduction interventions may be insufficient when delivered in isolation. Standard mental health interventions may overlook structural factors such as financial insecurity and household stress, which are associated with mental health problems^12,13^. Mental health problems may also manifest differently in the contexts of poverty and have different developmental pathways, suggesting management may benefit from economically informed approaches^14–16^. Similarly, poverty-reduction interventions alone may not be sufficient to generate sustained economic mobility or improve mental health outcomes. Mental health problems are associated with worse socioeconomic outcomes^17,18^and poverty-reduction interventions may not address the psychological or biological determinants of mental health outcomes^19,20^. Given these interdependencies, integrated approaches that address both economic and psychological dimensions may have greater and more long-lasting benefits than either approach alone^13^.

Despite growing evidence of the bidirectional relationship between poverty and mental health, existing reviews have focused on the impacts of either psychological or poverty-reduction interventions separately^21,22^. As a result key questions remain regarding the added value of combining psychological and poverty-reduction interventions, types of interventions being combined and their feasibility. This paper aims to systematically review the literature for poverty-reduction interventions that are combined with psychological interventions and to understand their impact on mental health and socioeconomic outcomes.

Objectives.

This review aims to characterise combined poverty-reduction and mental health interventions and to better understand:

(i) What types of poverty-reduction and psychological interventions are used in combination, and how are they delivered.
(ii) What populations are targeted and in which countries/settings are these interventions evaluated?
(iii) With regard to feasibility, what implementation challenges/solutions to combining interventions are investigated.
(iv) What mediators and moderators are investigated.
(v) What are the impacts of these interventions on mental health and socioeconomic outcomes.

## Results

**Fig. 1:**
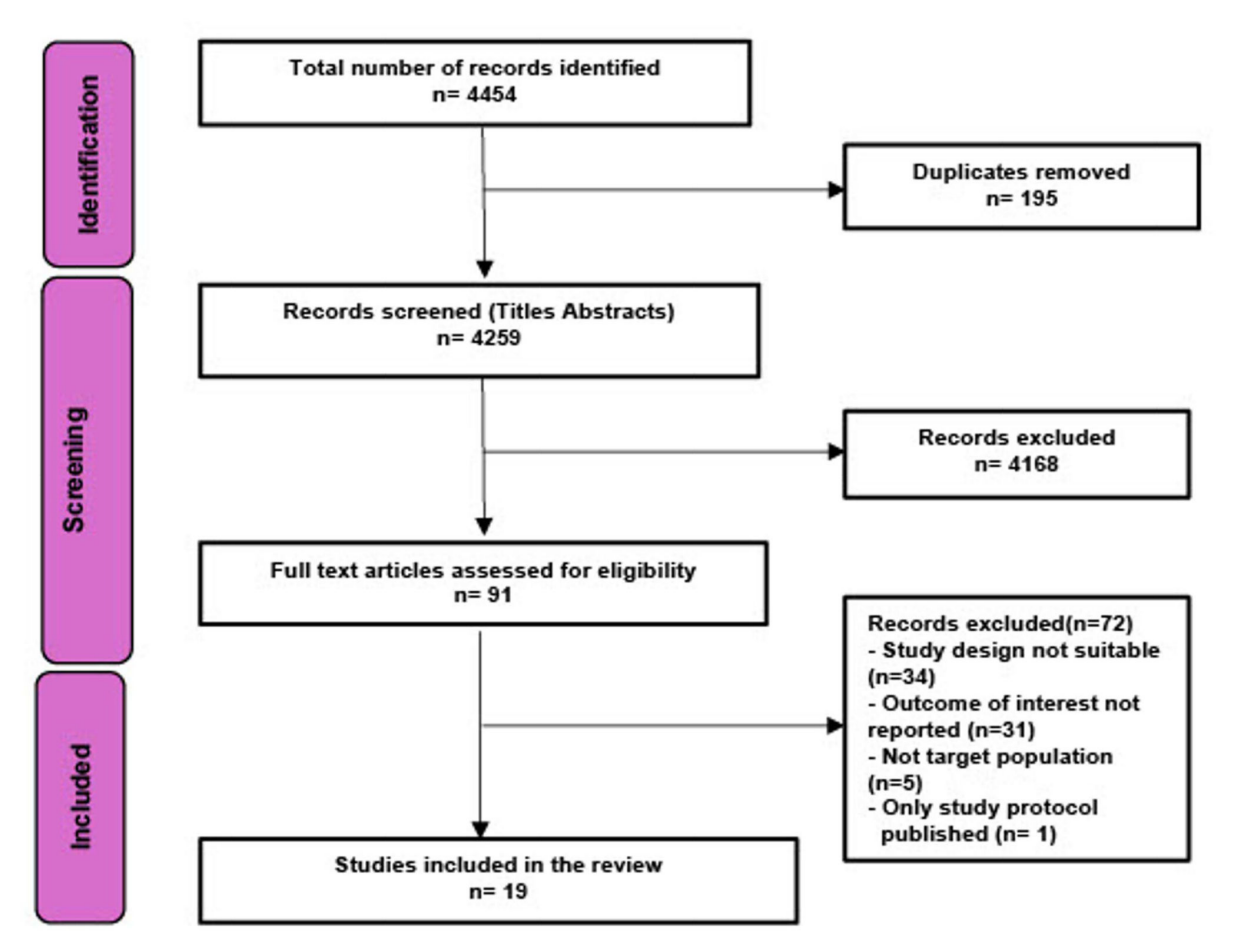
2020 PRISMA diagram.

Database searching, expert consultation and reference checking yielded 4,259 non-duplicate articles. 19 articles on 17 studies were included following full text review (Fig. 1)^23–42^. All included articles and detailed study descriptions are referenced in Supplementary Table 2.

### Participant characteristics and targets of interventions

Most studies (*n* = 10; 59%) focused on adults (two of which only included older adults, and two of which only included parents) while over a quarter (*n* = 5; 29%) targeted adolescents and young adults (ages 12–35). Two studies (12%) targeted families or parent child dyads. Most studies included participants of any sex and seven (42%) targeted females and two (12%) targeted males. Most interventions (*n* = 11; 65%) did not target a specific mental health condition. However, six (35%) studies targeted individuals with mental health difficulties, including four studies (17%) focused on people with depression, one study focused on depression and anxiety, and one study focused on suicide.

In relation to poverty, three studies targeted individuals who were beneficiaries of government assistance programmes (18%), seven (42%) included low-income or poor participants, two (12%) targeted individuals living in high-poverty areas, and two (12%) targeted individuals with self-reported financial difficulties. Studies also targeted specific vulnerable populations such as orphans (*n* = 2, 12%), young men engaged in crime and violence or poor and at-risk of engaging in such activities (*n* = 1, 6%). Six studies (35%) targeted participants based on both mental health and poverty related characteristics. Just over half of studies (*n* = 10; 59%) were carried out in a LMIC though a large proportion (*n* = 7; 41%) were carried out in high income settings (in the USA, UK and Hong Kong, see Table 1). See Supplementary Table 2 for full study details.

### Types of psychological/psychosocial interventions

The most common approach to delivering psychological and/or psychosocial interventions was delivery by a non-specialist professional (*n* = 8; 47%). Additional delivery modalities included life skills training or mentoring (*n* = 4; 24%), delivery by a mental health professional (*n* = 3, 18%) and digital platforms (*n* = 1, 6%). Intervention content varied across studies. Life skills training or mentoring approaches were used in five studies (29%). Among interventions that explicitly described a psychological framework, CBT was most common (*n* = 6; 35%), followed by interpersonal therapy (*n* = 2; 12%), motivational interviewing (*n* = 1; 6%), mindfulness-based approaches (*n* = 1; 6%), psychosocial stimulation (*n* = 1; 6%), resiliency theory (*n* = 1, 6%) and trauma-informed peer support (*n* = 1; 6%). Three studies (18%) did not specify a psychological theory.

### Types of poverty-reduction interventions

Studies included a wide variety of poverty-reduction strategies and policies, with several (*n* = 4, 24%) providing multiple interventions. These included: cash or asset transfer (*n* = 9, 53%); case management, including e.g., support with housing or employment (*n* = 4, 24%); debt or money advice (*n* = 3, 18%); savings groups (*n* = 3, 18%); government welfare/assistance benefits (*n* = 2, 12%); and vocational training (*n* = 2, 12%).

### Implementation challenges, mediators and moderators

Implementation challenges were often discussed post-hoc as potential explanations for null or modest effects (*n*= 5, 29%). The limited presentation of implementation challenges may be due to their reporting in separate qualitative publications^43^. Some studies in LMICs referenced issues related to limited resources and infrastructure. One study that tested a mentoring and cash transfer intervention to promote adolescent girls’ wellbeing highlighted the difficulties of addressing broader structural drivers such as early marriage and post-Ebola and post-conflict social conditions, as well as limited access to mental health or social services^38^. Another study conducted in India^36^ utilised a light-touch, phone-based CBT intervention combined with cash for older adults living alone. The authors suggest that the lack of mental health improvements may reflect limitations of phone-based delivery in building rapport and engaging older adults with limited experience using this modality. A study that combined culturally adapted interpersonal therapy with case management (including support for housing, childcare and transportation), reported limited participant adherence, despite efforts to offer a culturally adapted programme^31^, highlighting challenges in engaging low-income pregnant women.

Few studies explored potential mediators (*n* = 2, 12%) and moderators (*n*= 3; 18%). One study investigated mediators such as participant needs being met, engagement in pleasant activities, assertiveness and social support but did not find that any of these outcomes predicted depression scores^23^. Another study found that forward-looking time preferences and self-control skills mediated the combined intervention’s positive effects on antisocial behaviour^25^. The addition of case management to group CBT only improved depression symptoms and social functioning of Spanish speaking patients but not English-speaking patients in a small RCT. A large RCT in India found that all three treatment combinations (cash only, CBT-only, and cash combined with CBT) had beneficial impacts on mental health functioning post-intervention in women but not men^36^, suggesting that gender may be an important moderator for certain psychological outcomes. Similarly, another study found that cash transfers were associated with slightly higher expenditure in male recipient households and slightly lower revenue in households led by individuals with high psychological distress at baseline^32^. The CBT-based psychological component effects on the psychological wellbeing index (a weighted average of psychological distress, stress and disability) were not greater amongst participants with psychological distress at baseline.

### Impacts of combined intervention on mental health problems and substance use outcomes

Table 2 shows the results of 17 studies (100%) that examined effects of combined psychological and poverty-reduction interventions on mental health and substance use outcomes. Among studies comparing combined interventions to control groups, seven of 14 studies (50%) found improvements in at least one outcome. Comparing to poverty-reduction interventions alone (testing the marginal effect of the psychological intervention) 0 of seven studies (0%) found significant reductions. Comparing to psychological interventions only (testing the marginal effect of the poverty-reduction intervention), two of five (40%) found significant reductions (e.g. depression, perceived stress) and one of five (20%) found significant increases in mental health problems (e.g. child depression and child psychological distress).

Adult depression, anxiety, and distress were the most frequently assessed outcomes (11 studies). Of these, seven (64%) reported reductions in symptoms. Six of eleven (55%) found improvements when comparing combined interventions to control, and two of eleven (18%) found improvements when comparing combined interventions to psychological interventions alone. Five studies (29%) evaluated effects on other outcomes, including posttraumatic stress disorder (PTSD), antisocial behaviour, substance use, suicidal ideation and child mental health. Among these, only one (20%) found improvements.

**Fig. 2:**
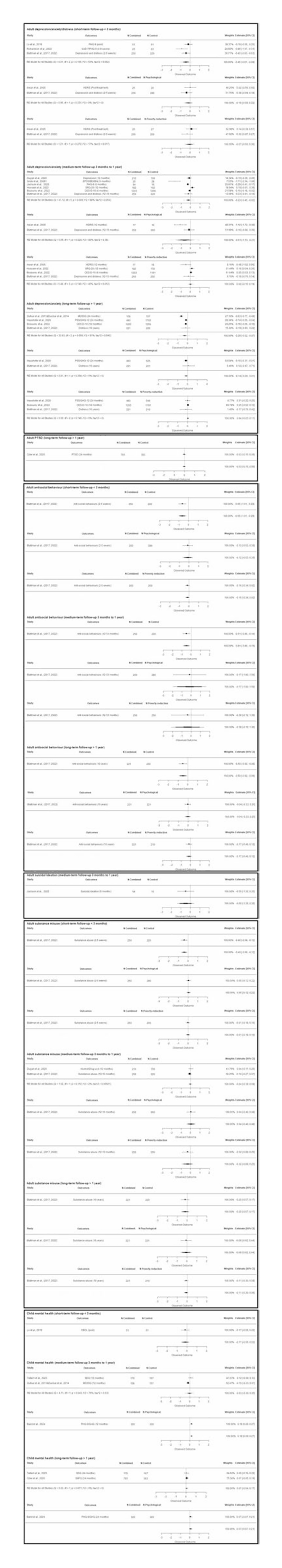
Forest plots of the effects of combined interventions on mental health outcomes. Forest plots are displayed by outcome subcategory, follow-up duration, and comparator. Effect sizes are represented by squares and solid lines (95% CIs). Negative effect sizes indicate a reduction in mental health symptoms.

Across studies comparing combined interventions to control, effect sizes ranged from − 1.73 to 0.18 (Fig. 2), with only one study finding increases in mental health problems or substance use^24^. Findings suggest that interventions incorporating an poverty-reduction component—whether alone or as part of a combined approach—were more effective than psychological interventions alone, particularly at short-term follow-up (less than three months). One notable exception to this pattern found an increase in symptoms with the addition of a poverty-reduction component^24^. See Supplementary Table 5 for a summary of intervention effects by outcome domain, comparator, and time point.

### Impacts of combined intervention on positive mental health/wellbeing outcomes and social functioning

Table 3 presents results of twelve studies (71%) that examined effects of combined psychological and poverty-reduction interventions on any positive mental health and wellbeing outcome, including psychological wellbeing, self-esteem, self-efficacy, and resilience.

Among studies comparing combined interventions to control, 4 of 7 studies (57%) found improvements in at least one outcome measure (e.g. positive self-regard, subjective wellbeing, inner peace, happiness). Compared to poverty-reduction interventions alone (estimating the marginal effect of the psychological intervention), one of eight studies (12.5%) found improvements; and compared to psychological interventions only (estimating the marginal effect of the poverty-reduction intervention), two of five (40%) found improvements and one of five (20%) found reductions in positive mental health and wellbeing (Table 7).

Psychological wellbeing was the most frequently assessed positive mental health outcome, assessed in 4 studies three of which (75%) reported positive effects of the combined interventions. Other outcomes showed less consistent effects, except for self-esteem for the combined versus control comparison at medium-term follow-up.

**Fig. 3:**
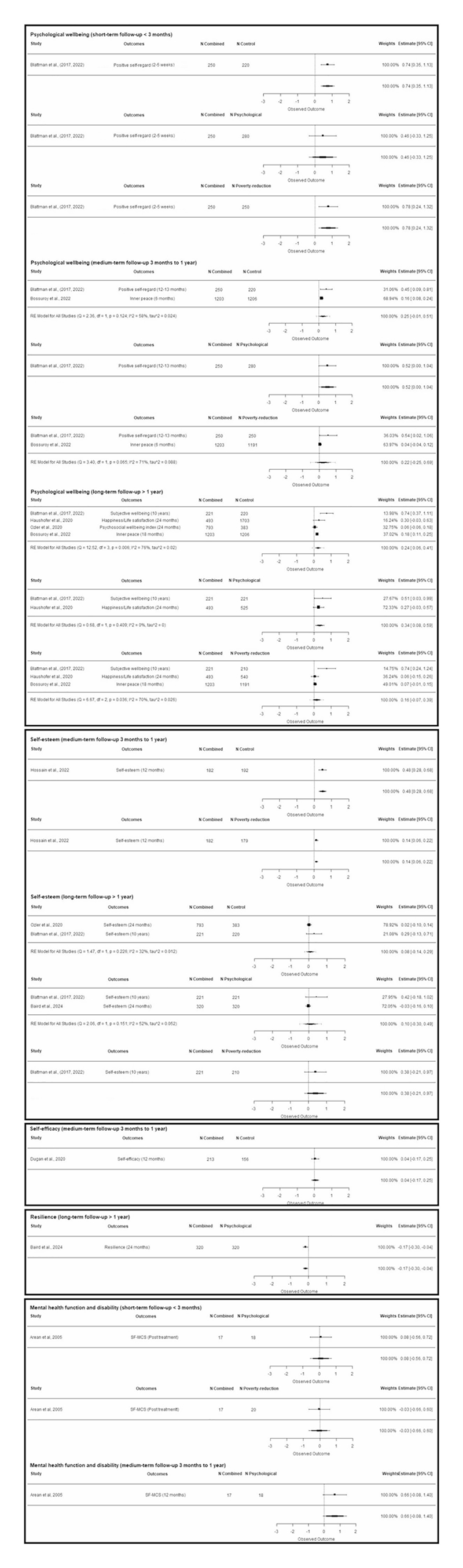
Forest plots of the effects of combined interventions on positive mental health/wellbeing. Forest plots are displayed by outcome subcategory, follow-up duration, and comparator. Effect sizes are represented by squares and solid lines (95% CIs). Positive effect sizes indicate an improvement in positive mental health/wellbeing.

Across all comparisons, effect sizes ranged from − 0.17 to 0.78 (Fig. 3). Only one study^24^ reported negative effects on positive mental health and wellbeing. These findings suggest combined interventions are more frequently associated with improvements in positive mental health and wellbeing than either component alone, particularly for psychological wellbeing outcomes and across all follow-up periods.

### Impacts of combined interventions on socioeconomic outcomes

Table 4 shows the results of ten studies (59%) that examined the effects of combined psychological and poverty-reduction interventions on any socioeconomic outcome, including financial self-efficacy/wellbeing, economic hardship, daily/monthly consumption, household/personal revenue, food security, economic performance, and employment.

Among studies comparing combined interventions to control groups, four out of eight studies (50%) found significant improvements in at least one socioeconomic outcome (i.e. food security, household assets, consumption, household revenue). Compared to psychological interventions alone (estimating the marginal effect of the poverty-reduction intervention), two out of four studies (50%) found significant improvements while one (25%) found negative impacts on resilience. Compared to poverty-reduction interventions only (estimating the marginal effect of the psychological intervention), three out of seven studies (43%) found significant improvements on socioeconomic outcomes (e.g. consumption, household revenue, economic hardship, and food security), while one of six studies (17%) found negative impacts on household sever food insecurity. The most frequently measured outcome was food security (*n* = 4, 40%), with statistically significant results in the long-term comparisons of combined versus control and combined versus poverty-reduction.

**Fig. 4:**
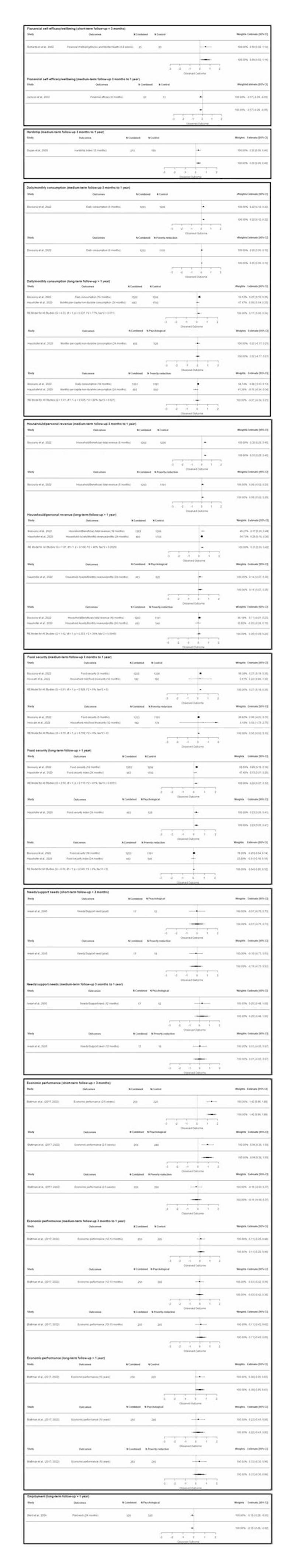
Forest plots of the effects of combined interventions on socioeconomic outcomes. Forest plots are displayed by outcome subcategory, follow-up duration, and comparator. Effect sizes are represented by squares and solid lines (95% CIs). Positive effect sizes indicate an improvement in socioeconomic outcomes. Data availability:.

Across all comparisons, effect sizes ranged from − 7.32 to 0.64 (Fig. 4), with two studies reporting a significant negative impact on socioeconomic outcomes (food security and probability of work). These findings suggest that the combined approach had more consistent positive impacts on socioeconomic outcomes than either psychological or poverty-reduction interventions alone, particularly when assessing outcomes at long-term follow-up (over one year). They also indicate the potential for negative marginal effects of psychological or poverty-reduction components within combined interventions.

### Quality assessment

Thirteen studies (76%) were assessed as having high quality (meeting at least five out of the six criteria outlined above) while four (24%) were assessed as having low quality (meeting less than four out of the six criteria outlined above) (see Supplementary Fig. 1).

## Discussion

This systematic review synthesised the evidence for interventions that combine poverty-reduction and psychological components and their impacts on mental health and socioeconomic outcomes. Across studies, the most common type of psychological interventions were psychological or psychosocial interventions delivered by a non-specialist professional. The most common poverty-reduction interventions were cash or asset transfers. While most interventions targeted adults or families, a significant minority (29%) focused on adolescents and young people. Combined psychological and poverty-reduction interventions were more consistently associated with improvements in mental health problems, positive mental health, and socioeconomic outcomes than either component alone. Even though positive mental health and wellbeing outcomes were slightly more likely to be observed at short-term follow-up, some effects persisted at medium-to long-term follow-up, suggesting potential for lasting benefits. Positive socioeconomic outcomes were more likely at long-term follow-up, suggesting improvements may take longer than mental health changes to materialise but could be sustained once achieved. While effects varied depending on the comparator, follow-up period, and outcome assessed, the clearest benefits were observed for adult depression, anxiety, and psychological wellbeing. Evidence for socioeconomic outcomes was more variable and appeared sensitive to study context and design. Nonetheless, across diverse outcomes and follow-up periods, combined interventions more often outperformed psychological interventions alone.

While our review suggests potential advantages of combined psychological and poverty-reduction interventions for improving mental health and socioeconomic outcomes, studies varied widely in terms of the context, the type and the duration of the intervention, making it difficult to compare and generalise findings. Heterogeneity in comparison groups was particularly relevant. Most studies (*n* = 14, 88%) included an arm that compared the combined intervention to inactive control, but fewer compared combined interventions to either psychological or poverty-reduction interventions alone (*n* = 7, 44%) and only three^26,32,36^ (19%) allowed for comparison across four treatment groups (control, poverty-reduction, psychological, and combined). This limits our ability to draw definitive conclusions about the added value of combining intervention components beyond either approach alone. Finally, heterogeneity in social protection systems, service capacity and political economy across cultures and contexts limits the transportability of inferences and thus we interpret consistency of patterns across settings cautiously. We also emphasise that future interventions should consider adaptation according to contexts and including co-production with communities to increase programme relevance and sustainability.

Despite these methodological constraints, a distinct pattern emerged. Studies comparing combined interventions to control conditions reported positive effects most often, particularly for depression, anxiety and psychological well-being. Yet, few benefits to mental health problems or wellbeing were observed when combined interventions were compared to poverty-reduction interventions alone, suggesting that psychological components did not consistently improve mental health or wellbeing beyond that achieved by the poverty-reduction components. Notably, no studies found positive effects on mental health problems when comparing combined interventions to poverty-reduction interventions, and only two reported improvements in well-being outcomes. The limited added benefit of psychological components could reflect at least two possibilities. First, adding a psychological component may yield diminishing returns if proximal stressors such as food insecurity or income instability are the dominant concern and addressed sufficiently by the poverty-reduction component. Second, many psychological interventions were brief and may have lacked the intensity or fidelity necessary to achieve meaningful effects. In such cases, null findings may reflect implementation challenges rather than a lack of therapeutic value.

Only one study found consistent negative effects across mental health, wellbeing and socioeconomic outcomes^24^. They reported negative marginal effects of an unconditional cash transfer at the end of a fourteen-week group interpersonal therapy intervention for adolescent girls in Uganda. Participants reported frustration at being unable to use the cash for planned purposes, possibly because of receiving the benefit during the COVID-19 pandemic, a period of prolonged school closures and restricted opportunities. Instead, participants reported needing to use the cash for essential needs for themselves and their family members. These results underscore the importance of context in determining how recipients perceive poverty-reduction interventions and in turn, how their mental health and wellbeing are impacted. They also suggest that poverty reduction interventions that do not change recipients’ material circumstances are unlikely to improve their mental health.

Indeed, among the five studies that reported positive socioeconomic outcomes, all but one^30^ found improvements in mental health or wellbeing (if reported). In most of these cases, the poverty-reduction component was substantial^25–27,32^. Our ability to draw conclusions about the relationship between mental health and socioeconomic outcomes is limited by the large proportion of studies (39%) that did not report socioeconomic outcomes, and the lack of relevant mediation analyses conducted. However, this co-occurrence suggests that poverty-reduction interventions’ positive mental health and wellbeing effects may be predicated on positive socioeconomic effects. This hypothesis is supported by prior literature. A meta-analysis found larger cash transfer values were associated with greater mental health effects^7^, and the Great Smoky Mountains study found that a yearly cash transfer programme was associated with mental health improvements in children lifted out of poverty, but not in children who remained poor^44^. These findings, along with the negative effects finding^24^ described above, suggest that mental health improvements may depend on the extent to which poverty-reduction interventions produce meaningful and sustained economic circumstances. Combined interventions may require both robust economic components and implementation within a context that allows for socioeconomic improvements to produce positive effects on mental health and wellbeing. Future research investigating combined poverty reduction and psychological interventions should examine the potential mediating role of socioeconomic effects on mental health outcomes and consider the broader structural and contextual conditions that may influence whether economic gains can be translated into mental health improvements.

Contextual factors likely shape the effectiveness, uptake and sustainability of combined interventions. These may include the availability and quality of existing mental health and social services^36,45^, social and cultural norms (for example, levels of stigma towards people with mental illness and living in poverty)^4,46^, and the political and economic conditions^47^ that determine existing opportunities for the target population including welfare architecture and informality of labour markets. These contextual factors vary widely throughout the different countries in which the included studies were conducted, further complicating our ability to draw conclusions about the role of context. Only four studies (24%) discussed implementation challenges, mostly within the context of post-hoc explanations of null effects. Reported barriers included resource constraints, an inability to change broader infrastructure, and low treatment engagement may have contributed to a lack of positive impacts. Our findings caution against universal prescriptions and instead highlight the importance of situating interventions within their structural and socio-political contexts, using measurement strategies that capture these influences, and prioritising co-production with communities to ensure both relevance and sustainability.

Our review identified several gaps in the literature that should be addressed by future research. First, few studies (*n*= 5) that focused on childhood or adolescent outcomes, despite evidence that poverty and mental health problems have a profound and lasting impact on mental health and future life chances^48,49^. The Family Stress Model posits that household economic conditions influence child development via parental psychological distress^50^and recent research demonstrates that families with more resources experience attenuated family stress processes^51^. Interventions including poverty-reduction components may therefore be particularly effective in addressing child and youth mental health. Second, assessment of externalizing outcomes and substance use, which are often associated with poverty were underrepresented^52,53^; only four studies included such outcomes. Third, studies led by mental health or public health professionals were less likely to evaluate socioeconomic outcomes compared to those led by economists or social scientists. There was also a tendency for studies not led by mental health professionals to use unvalidated or composite measures of mental health or wellbeing which may have lacked reliability and cultural validity. Finally, few studies implemented interventions that combined specific and targeted psychological components with robust economic components, possibly because few studies were run by an interdisciplinary team. These gaps highlight the need for greater integration between the fields of mental health and economics, in the design, implementation and evaluation of combined interventions.

Given the early nature of studying multi-sectoral interventions in global mental health, we included studies that covered a wide variety of combined interventions, populations, settings, and outcomes. Although our findings provide insight about the potential for combining psychosocial and poverty-reduction interventions to reduce mental health problems substance use and to improve positive mental health/wellbeing and socioeconomic outcomes, our search was only performed in English (though we did not exclude any studies based on language) and so some relevant non-English studies may have been missed. Experts were consulted, and publications of relevant systematic reviews were hand searched but some may still have been missed. The studies we identified were heterogeneous in terms of the intervention approach, target population and study quality making it difficult to broadly generalise our findings. Populations studied, for example, included: low-income older adults, children or adolescents orphaned by AIDS, families receiving social welfare benefits, individuals with mild to moderate depression and anxiety etc. The relatively small number of included studies combined with their methodological heterogeneity limited our ability to draw conclusions about program effectiveness.

Our review suggests that combining psychological and poverty-reduction interventions can improve mental health and, to a lesser degree, socioeconomic outcomes, especially when compared to control. Across studies, the poverty-reduction components appeared to have more consistent positive effect on mental health problems and socioeconomic outcomes than the psychological components, especially when they offered substantial or sustained support. More research is needed to determine the optimal design, delivery, and evaluation of combined interventions, and to understand how they are shaped by contextual, structural and implementation-related factors. More studies that compare combined interventions with single-component arms are needed to disentangle the contribution of each component and their synergy. Additionally, more studies that focus on children and adolescents, assess externalizing outcomes, involve interdisciplinary teams and evaluate robust poverty-reduction interventions combined with targeted mental health interventions will advance the evidence base. This review also has implications for intersectoral care and potential approaches for addressing the complex and interrelated needs of people living in poverty and experiencing mental health problems. However, intersectoral approaches require overcoming many challenges, such as lack of resources, conflicting priorities, and poor communication. Given growing policy interest in multi-sectoral strategies that address both mental health and poverty-related risks future research needs to improve population mental health and reduce inequalities^54^.

## Methods

We searched MEDLINE (via OVID), PsycINFO (Via OVID), GLOBAL HEALTH (via Ovid), CINAHL (Via EBSCO) and ECONLIT (Via EBSCO) in English for articles related to poverty and mental health. We ran the search from January 1990 until February 2025 to align with the initiation and scaling of global poverty-alleviation programmes, including emergence of cash transfers. Databases were searched using database specific keywords and subject headings (See Supplementary Fig. 1). Additionally, lateral search techniques were used including: scanning reference lists (including from identified relevant reviews), searching keywords on Google Scholar and expanding the search via the ‘cited by’ option in PubMed. Corresponding authors of included studies were contacted for further recommendation. This review was registered with OSF^55^ and methods are reported in line with PRISMA guidelines (Fig. 1). No protocol was prepared. Two co-authors (SS, SEL) independently screened titles and abstracts, followed by full-text review. To ensure reliability, 10% of studies were randomly selected for double screening. Discrepancies were resolved through discussion, and inclusion/exclusion criteria were refined iteratively. All included studies were re-screened against final eligibility criteria. SS then completed the remaining screening independently. Five reviewers then recorded study characteristics on a shared spreadsheet using a pre-piloted data extraction form. To ensure consistency, data from the first 10% of studies were extracted in pairs. The remaining studies were extracted individually.

### Inclusion and exclusion criteria

Inclusion and exclusion criteria were specified in advance and refined following piloting. Given the limited prior evidence on combined mental health and poverty-reduction interventions, we purposively adopted a broad approach to inclusion.

(1) *Study design*. We included articles with experimental or quasi-experimental evaluation designs, including prospective and retrospective cohort studies, and randomised and non-randomised evaluations. We excluded reviews, editorials, commentaries, non-empirical papers, or those using only qualitative methods.
(2) *Population*: We considered individuals/households/communities living in poverty, defined broadly in line with the Multidimensional Poverty Index (MPI), encompassing deprivations in education, health and living standards^56^. Studies did not need to use the MPI for inclusion nor did we quantify MPI values. We included clinical and non-clinical populations aimed at mental health prevention, promotion or treatment. We included mental health and substance use-related conditions as defined by DSM-V.
(3) *Type of intervention*. Studies had to include both a poverty-reduction and psychological/psychosocial component. We included poverty-reduction components which aimed to improve material circumstances or provide resources and services to enhance economic stability. These included cash or asset transfers, in-kind benefits (e.g., food, housing) or services (e.g., financial coaching, case management).
  For psychological/psychosocial components, we included those with a stated aim around mental health treatment, prevention or promotion and that articulated mental health mechanisms or outcomes in their theory of change or aims. We included any modality, i.e., psychological, psychosocial and pharmacological modalities. We excluded: (i) Interventions only addressing physical health; (ii) Psychological interventions without an poverty-reduction component; (iii) Poverty-reduction interventions without a psychological component; (iv) Financial incentives to quit smoking/alcohol/attend therapy in populations other than those living in poverty; (V) Reimbursements for study participation or for travel to an evaluation; (vi) studies recruiting individuals based on receiving government benefits without a specific aim to assess its effect on mental health or socioeconomic outcomes.
(4) *Comparison groups*: We included studies that compared combined poverty-reduction and psychological interventions to: poverty-reduction interventions alone; psychological interventions alone, or standard care, usual services or no intervention.
(5) *Outcomes*. Our review included studies that reported at least one assessment of mental health or socioeconomic-related outcomes; studies were not required to report both for inclusion. We included a broad range of mental health outcomes including symptoms of common and severe mental disorders in alignment with the DSM-V as well as psychological distress and suicidality, as reported by participants, clinicians or other informants. We also considered indicators of positive mental health and wellbeing. Outcomes could be assessed using either categorical or continuous scales. In relation to socioeconomic outcomes, we considered any indicators of the material and social conditions of study participants. This includes, for example, education, employment, earnings, consumption, wealth, occupation, social class or social mobility.

### Quality assessment

Six quality criteria were used and assessed at the time of data extraction. Criteria used were adapted from the Evidence for Policy and Practice Information and Co-ordinating Centre. These criteria are: (1) aims clearly stated, (2) design appropriate to the stated objectives, (3) justification given for sample size, (4) evidence provided of reliability or validity of measures used, (5) statistics were accurately reported and (6) sample selection was relatively unbiased.

### Data extraction and synthesis

Data were extracted using a standardised, pre-piloted form. Missing data were requested from study authors. Detailed information was extracted on study design, participant characteristics, study context, intervention details and components, control/comparison conditions, timing of follow-ups, outcomes and any potential mediators and moderators assessed. We extracted means and standard deviations of continuous outcomes to calculate Hedges’ g. Where studies reported odds ratios, regression coefficients or other estimates, values were converted to Hedge’s g using the Campbell Collaboration effect size calculator^57^.

### Analysis

We reported the outcome measure, baseline and follow-up summary statistics, standardised effect size estimate and 95% confidence interval for each relevant outcome (mental health problems and substance use, positive mental health and wellbeing outcomes and socioeconomic outcomes). We synthesised outcomes by outcome type, follow-up duration and comparator. We categorised results into three broad outcome categories; (1) mental health and substance use outcomes, 2) positive mental health and wellbeing outcomes and 3) socioeconomic outcomes. Outcome types were further divided into subcategories such as adult depression and anxiety, child mental health, and adult substance use within the mental health and substance use category, or economic performance and food security within the socioeconomic outcome category. Results were further divided by follow-up duration: short-term (less than three months), medium-term (three months to one year), and long-term (over one year), and comparator (combined versus psychosocial intervention only, combined versus poverty-reduction intervention only, or combined versus control). In cases where all participants were enrolled in a government benefits program as part of their eligibility criteria, any psychological or additional economic intervention was considered an added component rather than a standalone poverty-reduction intervention.

Past meta-analyses of poverty-reduction interventions have taken a variety of approaches when handling different follow-up periods, from simply choosing the longest follow-up and meta-analysing studies with a wide range of follow-up periods^5^ or including subsequent follow-ups as an individual study in the meta-analysis^7^. Given the high degree of clinical and methodological heterogeneity in this review, we did not conduct a meta-analysis with a single pooled summary effect. We instead calculated pooled effect sizes within relatively comparable subgroups to visually display the distribution of effects. Studies without sufficient data to calculate Hedge’s g values were excluded from the quantitative synthesis but included in the narrative synthesis. A summary table of intervention effect directions by outcome domain, comparator, and time point was created to visually display all included study results.

We used forest plots to provide a quantitative summary of effect sizes and displayed effect estimates by outcome. Pooled effect sizes were conducted for each outcome domain, stratified by outcome subcategory (e.g. adult depression and anxiety), follow-up duration, and comparator. We used bias-corrected Hedge’s g to adjust for small sample size bias. When multiple outcomes or follow-up times were reported within the same subgroup, we selected a single estimate using the following criteria: the longest follow-up, the most comprehensive outcome (e.g., total rather than subscale scores), or the median effect size (e.g., when both the Patient Health Questionnaire-9 (PHQ-9) and the Generalized Anxiety Disorder-7 (GAD-7) were reported).

## Supplementary Material

**Supplementary Information** The online version contains supplementary material available at https://doi.org/10.1038/s41598-025-24736-8.

Supplementary Material 1

## Figures and Tables

**Table 1:** Intervention Characteristics.

Study	Country/context	Target group	Mental health intervention modality	Psychological theory informing the mental health intervention	Treatment, prevention, promotion	Poverty-reduction intervention category	Control description	Treatment groups
			(1) psychotherapy delivered by a mental health professional; (2) life skills training/mentoring (3) psychosocial or psychological intervention delivered by a non-specialist professional; (4) digital cognitive behavioural therapy(5) mindfulness			(1) government welfare/assistance benefits; (2) debt or money advice; (3) cash or asset transfer; (4) microfinance; (5) savings group; (6) case management; (7) vocational training		
Arean et al., 2005	USA	Low-income older adults > 60 yrs with with depression	Psychotherapy delivered by a mental health professional	CBT	Treatment	Case management	NA (either psychological or antipoverty as comparators)	Psych +APPsychAP
Baird et al., 2024	Uganda	Adolescent girls (13–19 years) with depression in high poverty areas	Psychosocial or psychological intervention delivered by a non-specialist professional	IPT	Treatment	Cash or asset transfer	Usual care	Psych + APPsychControl (no treatment)
Blattman et al., (2017, 2022)	Liberia	18–35 year high-risk men who engaged in part-time theft and drug dealing and regularly had violent confrontations	Psychosocial or psychological intervention delivered by a non-specialist professional	CBT	Treatment	Cash or asset transfer	No treatment	Psych +APPsychNo treatment
Bussoroy et al., 2022	Niger	Extremely poor female beneficiaries already enrolled in a national cash transfer government programme	Life skills training/mentoring	Social and cultural psychology theories of behavior change (?)	Promotion	Debt or money advice, cash or asset transfer, savings group	Government antipoverty programme	Psych + AP Control (government antipoverty programme)*
Dufour et al., 2011&Dunbar et al., 2014	Zimbabwe	Out-of-school orphan girls ages 16 to 19 years	Life skills training/mentoring	None	Prevention and promotion	Vocational training; cash or asset transfer	NA (psychological intervention as comparator)	Psych + APPsych
Dugan.et al., 2020	USA (Philadelphia)	Parents receiving social welfare benefits	Psychosocial or psychological intervention delivered by a non-specialist professional	Trauma-informed peer support	Treatment	Government welfare/assistance benefits; cash or asset transfer	Government antipoverty programme	Psych + APControl (government antipoverty programme)
Grote et al., 2009	USA	Pregnant, low-income women with depression	Psychotherapy delivered by a mental health professional	IPT	Treatment	Case management	Enhanced usual care	Psych + APControl (enhanced usual care)
Haushofer et al., 2020	Kenya	Adults within households living in a house without brick, stone, or metal walls.	Psychosocial or psychological intervention delivered by a non-specialist professional	CBT	Treatment	Cash or asset transfer	No treatment	Psych + APPsychAPControl (no treatment)
Hossain et al., 2022	Bangladesh	Poor mother and child (6–16 months) dyads	Psychosocial or psychological intervention delivered by a non-specialist professional; life skills training/mentoring	Psychosocial stimulation	Prevention and promotion	Cash or asset transfer	No treatment	Psych + APAPControl (no treatment)
Jackson et al., 2022	UK (Bristol, North Somerset and South Gloucestershire area)	Men aged 30–64 who are suicidal and also have debt, financial, employment or welfare difficulties	Psychosocial or psychological intervention delivered by a non-specialist professional	MI	Treatment	Debt or money advice	Low-exposure	Psych + APControl (low-exposure)
Lo et al., 2019	Hong Kong	Families receiving social welfare benefits	Psychosocial or psychological intervention delivered by a non-specialist professional	Mindfulness	Promotion	Government welfare/assistance benefits	Waitlist	Psych + APAP
McKelway et al., 2023	India	Very poor adults 55+, living alone	Psychosocial or psychological intervention delivered by a non-specialist professional	CBT	Treatment	Cash or asset transfer	No Treatment	Psych + APPsychAPControl (no treatment)
Miranda et al., 2003a	USA	Low-income Black and Latina women with current major depression	Psychotherapy delivered by a mental health professional	CBT	Treatment	Case management	NA (psychological intervention as comparator)	Psych + APPsych
Ozler et al., 2020	Liberia	Adolescent girls (13–14 years) in a high-poverty, rural county	Life skills training/mentoring	None	Prevention	Vocational training; Savings groups	No treatment	Psych + APControl (no treatment)
Richardson et al., 2022	UK	Adults with mild to moderate depression or anxiety and financial stress	Digital platform	CBT	Treatment	Debt or money advice	No treatment (Pre vs post)	Psych + AP (post) Control (pre)
Roelen et al., 2021	Haiti	Women living in extreme poverty	Psychosocial or psychological intervention delivered by a non-specialist professional	None	Prevention and promotion	Cash or asset transfer; Saving groups; Case management	No treatment (matched)	Psych + APControl (no treatment)
Ssewamala et al., 2012; Tutlam et al., 2023	Uganda	Children 12–16 years of age orphaned by AIDS	Life skills training/mentoring	Resiliency theory	Promotion	Microfinance; Savings groups	NA (includes psychological component)	Psych + APControl (enhanced usual care)

**Table 2:** Relationship between study intervention(s) and mental health and substance use outcomes. Statistically significant improvements are colored in green, null in grey, and statistically significant worsening in red

Study reference	Outcome measure	Combined vs. control		Combined vs. psychological		Combined vs. poverty-reduction	
		Bias corrected effect size (Hedges)	95% CI	Bias corrected effect size (Hedges)	95% CI	Bias corrected effect size (Hedges)	95% CI
Adult depression/anxiety/distress (short-term follow-up <3 months)							
Lo et al., 2019	PHQ-9 (post)					–0.18	–0.57, 0.20
Arean et al., 2005	HDRS (Post treatment)			0.02	–0.56, 0.60	0.14	–0.40, 0.67
Richardson et al., 2022	GAD-7 (4-8 weeks)	–0.69	–1.23, –0.15				
Richardson et al., 2022	PHQ-9 (4-8 weeks)	–1.09	–1.71, –0.47				
Blattman et al., (2017, 2022)	Depression and distress (2-5 weeks)	–0.43	–.83, –0.02	–0.38	–0.94, 0.18	–0.30	–0.87,0.28
Adult depression/Anxiety (medium-term follow-up 3 months to 1 year)							
Arean et al., 2005^3^	HDRS (6 months)			–0.2	–0.78, 0.38	–0.01	–0.55, 0.52
Arean et al., 2005	HDRS (12 months)			–1.10	–1.73, –0.48	–0.48	–1.03, 0.06
Dugan et al., 2020	Depression (0-12 months)	–0.15	–0.36, 0.06				
Grote et al., 2009^1,3^	EPDS (3 months)	–1.35	–1.95, –0.76				
Grote et al., 2009^1,3^	BDI (3 months)	–1.15	–1.73, –0.58				
Grote et al., 2009^1,3^	BAI (3 months)	–1.25	–1.83, –0.66				
Grote et al., 2009^1^	EPDS (9 months)	–2.02	–2.65, –1.33				
Grote et al., 2009^1^	BDI (9 months)	–1.78	–2.39, –1.12				
Grote et al., 2009^1^	BAI (9 months)	–1.34	–1.94, –0.74				
Jackson et al., 2022	PHQ-9 (6 months)	–0.29	–0.41, –0.17				
** Hossain et al., 2022	SRQ-20 (12 months)	0.19	–0.01, 0.39			0.16	–0.04, 0.37
Bossuroy et al., 2022^+^*	CES-D-10 (6 months)	–0.10	–0.03, –0.19			0.05	–0.03, 0.14
Blattman et al., (2017, 2022)	Depression and distress (12–13 months)	–0.23	–0.61, 0.15	–0.16	–0.69, 0.36	–0.18	–0.71, 0.34
Adult depression/Anxiety (long-term follow-up > 1 year)							
Dufour et al., 2011&Dunbar et al., 2014^3^	Mental Distress Shona Symptom Questionnaire (18 months)	–0.02	–0.36, 0.32				
Dufour et al., 2011&Dunbar et al., 2014	Mental Distress Shona Symptom Questionnaire (24 months)	–0.63	–1.082, –0.178				
Haushofer et al., 2020	Perceived Stress Scale (Endline, 24 months)	–0.22	–0.32, –0.12	–0.22	–0.37, –0.07	–0.07	–0.22, 0.08
Haushofer et al., 2020	GHQ12 (24 months)	–0.07	–0.19, 0.05	–0.10	–0.26, 0.06	0.1	–0.06, 0.26
Bossuroy et al., 2022^+^*	CES-D-10 (18 months)	–0.18	–0.10, –0.26			0.05	–0.02, 0.13
Blattman et al., (2017, 2022)2	Distress (10 years)	–0.19	–0.60, 0.22	0.12	–0.47, 0.71	–0.17	–0.76, 0.42
Roelen et al., 2021^‡^	K6 (22 months)	–0.95	–1.10, –0.79^‡^				
Adult PTSD (long-term follow-up > 1 year)							
Ozler et al., 2020	Suffering from PTSD (24 months)	–0.03	–0.16, 0.09				
Adult antisocial behaviour (short-term follow-up <3 months)							
Blattman et al., (2017, 2022)	Anti-social behaviours (2-5 weeks)	–0.65	–1.01, –0.28	–0.12	–0.63, 0.39	–0.16	–0.34, 0.02
Adult antisocial behaviour (medium-term follow-up 3 months to 1 year)							
Blattman et al., (2017, 2022)	Anti-social behaviours (12-13 months)	–0.51	–0.86, –0.15	–0.17	–1.90, 1.57	–0.38	–2.12, 1.36
Adult antisocial behaviour (long-term follow-up > 1 year)							
Blattman et al., (2017, 2022)	Anti-social behaviours (10 years)	–0.5	–0.92, –0.08	–0.044	–0.335, 0.247	–0.168	–0.460, 0.124
Adult suicidal ideation (medium-term follow-up 3 months to 1 year)							
Jackson et al., 2022	Suicidal ideation (6 months)	–0.5	–1.26, 0.45				
Adult substance misuse (short-term follow-up <3 months)							
Blattman et al., (2017, 2022)	Substance abuse (2–5 weeks)	–0.40	–0.69, –0.12	0.05	–0.13, 0.22	–0.01	–0.19, 0.16
Adult substance misuse (medium-term follow-up 3 months to 1 year)							
Dugan et al., 2020	Alcohol consumption (0–12 months)	0.03	–0.18,0.24				
Dugan et al., 2020	Drug use (0–12 months)	0.05	–0.16,0.25				
Blattman et al., (2017, 2022)	Substance abuse (12–13 months)	–0.1	–0.28, 0.07	0.04	–0.41, 0.48	–0.32	–0.75, 0.12
Adult substance misuse (long-term follow-up > 1 year)							
Blattman et al., (2017, 2022)	Substance abuse (10 years)	–0.20	–0.58, 0.17	–0.09	–0.62, 0.44	^–0J1^	–0.78, 0.40
Child mental health (short-term follow-up <3 months)							
Lo et al., 2019	CBCL mental health (post)					–0.17	–0.56, 0.22
Lo et al., 2019	CBCL depression anxiety (post)					–0.11	–0.49, 0.28
Lo et al., 2019	CBCL depression (post)					–0.09	–0.48, 0.29
Lo et al., 2019	CBCL somatic complaint (post)					–0.11	–0.50, 0.28
Lo et al., 2019	CBCL attention (post)					–0.41	–0.80, –0.02
Lo et al., 2019	CBCL agression (post)					0.03	–0.36, 0.42
Lo et al., 2019	CBCL internalising (post)					–0.10	–0.49, 0.29
Lo et al., 2019	CBCL externalising (post)					–0.08	–0.47, 0.31
Child mental health (medium-term follow-up 3 months to 1 year)							
Ssewamala et al., 2012/Tutlam et al., 2023**	CDI (10 months)					–0.60	–0.84, –0.35
Dufour et al., 2011&Dunbar et al., 2014^3^	Mental Distress Shona Symptom Questionnaire (6 months)	–0.09	–0.37, 1.19				
Dufour et al., 2011&Dunbar et al., 2014	Mental Distress Shona Symptom Questionnaire (12 months)	–0.16	–0.060, 0.282				
Ssewamala et al., 2012/Tutlam et al., 2023**	SDQ (12 months)	0.12	–0.09, 0.33				
Baird et al., 2024	PHQ-9 (12 months)			0.18	0.10, 0.26		
Baird et al., 2024	GHQ (12 months)			0.18	0.07, 0.28		
Child mental health (long-term follow-up > 1 year)							
Ssewamala et al., 2012/Tutlam et al., 2023**	CDI (20 months)	–0.69	–0.91, –0.48				
Ssewamala et al., 2012/Tutlam et al., 2023**	SDQ (24 months)	0.05	–0.10, 0.32				
Ozler et al., 2020	SMFQ (24 months)	0.07	–0.05, 0.19				
Baird et al., 2024	PHQ-9 (24 months)			0.079	–0.07, 0.23		
Baird et al., 2024	GHQ (24 months)			0.06	–0.07, 0.20		

1 focus on perinatal depression.

2 Blattman reported outcomes differently at different follow-ups. The depression and distress index was not reported at the 10 year follow-up. Depression results did not provide the standard deviation of the dependent variable needed to calculate Hedge’s g from unstandardized OLS results, so only the distress outcome was extracted.

3 Excluded from meta-analysis due to repeated assessments within the same follow-up period.

* Only provided SD of the baseline control group, so that was used for the SD of the dependent variable.

** Used model-based f -tests.

‡‡ Cohen’s d provided, converted to Hedge’s g.

+ Reported results for the marginal effects of antipoverty or psychological interventions rather than the effect of each treatment versus control.

‡ Roelen did not include enough data accurately take clustering into account so these results are not included in the meta-analysis. Note: We were unable to convert results for Miranda and McKelway to Hedge’s g, see Supplementary Table 2.

**Table 3:** Relationship between study intervention(s) and positive mental health/wellbeing outcomes. Statistically significant improvements are colored in green, null in grey, and statistically significant worsening in red.

Study reference	Outcome measure	Combined vs. Control		Combined vs psychological		Combined vs. poverty-reduction	
		Bias corrected effect size (Hedges)	95% CI	Bias corrected effect size (Hedges)	95% CI	Bias corrected effect size (Hedges)	95% CI
Psychological wellbeing (short-term follow-up <3 months)							
Blattman et al., (2017, 2022)	Positive self-regard (2–5 weeks)	0.74	0.35, 1.13	0.46	–0.6, 0.98	0.78	0.24, 1.331
Psychological wellbeing (medium-term follow-up 3 months to 1 year)							
Blattman et al., (2017, 2022)	Positive self-regard (12–13 months)	0.45	0.09, 0.81	0.52	0, 1.04	0.54	0.01, 1.06
Bossuroy et al., 2022^+^	Inner peace (6 months)	0.16	0.07, 0.24			0.04	(–0.04, 0.13)
Psychological wellbeing (long-term follow-up > 1 year)							
Blattman et al., (2017, 2022)**	Subjective wellbeing (10 years)	0.74	0.37, 1.12	0.51	0.02, 0.99	0.74	0.24, 1.25
Haushofer et al., 2020	Happiness (24 months))	0.14	0.003, 0.28	0.10	–0.10, 0.30	–0.06	–0.24, 0.12
Haushofer et al., 2020	Life satisfaction (24 months)	0.45	0.35, 0.56	0.44	0.20, 0.68	0.17	–0.07, 0.41
Ozler et al., 2020	Psychosocial wellbeing index (24 months)	0.06	–0.06, 0.19				
Bossuroy et al., 2022^+^	Inner peace (18 months)	0.18	0.11, 0.25			0.07	(–0.01, 0.15)
Self-esteem (medium-term follow-up 3 months to 1 year)							
Hossain, et al., 2022*	Self esteem (12 months)	0.48	0.28, 0.69			0.14	–0.06, 0.35
Self-esteem (long-term follow-up > 1 year)							
Ozler et al., 2020	Self-esteem (24 months)	0.02	–0.10, 0.14				
Blattman et al., (2017, 2022)**	Self-esteem (10 years)	0.29	–0.13, 0.71	0.42	–0.17, 1.03	0.38	–0.22, 0.97
Baird et al., 2024	Self esteem (24 months)			–0.03	–0.16, 0.10		
Self-efficacy (medium-term follow-up 3 months to 1 year)							
Dugan et al., 2020	Self-efficacy (0–12 months)	0.04	–0.17, 0.25				
Resilience (long-term follow-up > 1 year)							
Baird et al., 2024	Resilience (24 months)			–0.17	–0.30, –0.04		
Mental health function and disability (short-term follow-up <3 months)							
Arean et al, 2005	SF-MCS (post)			0.08	–0.57, 0.72	–0.03	–0.67, 0.60
Mental health function and disability (medium-term follow-up 3 months to 1 year)							
Arean et al., 2005^3^	SF-MCS (6 months)			0.12	–0.64, 0.89	–0.15	–0.82, 0.52
Arean et al, 2005	SF-MCS (12 months)			0.66	–0.08, 1.40	0.00	–0.64, 0.64

* Cohen’s d provided, converted to Hedge’s g.

** Blattman reported outcomes differently at different follow-ups. The positive self-regard index was not reported at the 10 year follow-up so the subjective well-being and self-esteem outcomes were extracted.

+ Reported results for the marginal effects ofpoverty-reduction or psychological interventions rather than the effect of each treatment versus control.

3 Excluded from meta-analysis due to repeated assessments within the same follow-up period.

4 Excluded from meta-analysis as multiple conversions using the baseline standard deviation resulted in effect sizes not within the 95% confidence interval. Note: We were unable to convert results for McKelway to Hedge’s g, see Supplementary Table 3.

**Table 4:** Relationship between study intervention(s) and socio-economic outcomes. Statistically significant improvements are colored in green, null in grey, and statistically significant worsening in red.

Study reference	Outcome measure	Combined vs. control		Combined vs. psychological		Combined vs. poverty-reduction	
		Bias corrected effect size (Hedges)	95% CI	Bias corrected effect size (Hedges)	95% CI	Bias corrected effect size (Hedges)	95% CI
Fianancial self-efficacy/wellbeing (short-term follow-up <3 months)							
Richardson et al., 2022	In Charge Financial Distress/Financial Wellbeing scale (4-8 weeks)	0.64	0.05, 1.22				
Richardson et al., 2022	The Money and Mental Health Scale (4-8 weeks)	0.52	–0.06, 1.01				
Fianancial self-efficacy/wellbeing (medium-term follow-up 3 months to 1 year)							
Jackson et al., 2022	Financial efficacy FSES (6 months)	–0.17	–0.3,–0.05				
Hardship (medium-term follow-up 3 months to 1 year)							
Dugan et al., 2020	Hardship index (0-12 months)	0.26	0.06, 0.47				
Daily/Monthly consumption (medium-term follow-up 3 months to 1 year)							
**Bossuroy et al., 2022	Daily consumption (6 months)	0.22	0.12, 0.33			0.05	0.00, 0.10
Daily/Monthly consumption (long-term follow-up > 1 year)							
**Bossuroy et al., 2022	Daily consumption (18 months)	0.25	0.15, 0.35			0.08	0.03, 0.13
Haushofer et al., 2020	Monthly per-capita non-durable consumption (24 months)	0.08	–0.05, 0.20	0.02	–0.17, 0.22	–0.15	–0.34, 0.05
Househould/personal revenue (medium-term follow-up 3 months to 1 year)							
**Bossuroy et al., 2022	Household total revenue (6 months)	0.29	0.18, 0.39			0.09	–0.01, 0.19
**Bossuroy et al., 2022	Beneficiary total revenue (6 months)	0.4	0.29, 0.51			0.09	–0.03, 0.21
Househould/personal revenue (long-term follow-up > 1 year)							
**Bossuroy et al., 2022	Household total revenue (18 months)	0.31	0.21, 0.41			0.13	0.03, 0.22
**Bossuroy et al., 2022	Beneficiary total revenue (18 months)	0.43	0.28, 0.57			0.08	–0.06, 0.22
Haushofer et al., 2020	Assets Household owns (24 months)	0.36	0.20, 0.52	0.32	0.11, 0.52	–0.05	–0.25, 0.14
Haushofer et al., 2020	Monthly Household revenue (24 months)	0.16	–0.004, 0.32	0.08	–0.13, 0.29	–0.08	–0.30, 0.14
Haushofer et al., 2020	Monthly Household profits (24 months)	–0.10	–0.28, 0.08	–0.08	–0.30, 0.14	–0.03	–0.27, 0.21
Food security (short-term follow-up <3 months)							
McKelway et al.,2023^4^	Food security (3 weeks)						
Food security (medium-term follow-up 3 months to 1 year)							
**Bossuroy et al., 2022	Food security (6 months)	0.27	0.18, 0.35			0.06	–0.04, 0.15
*Hossain, et al., 2022	Household mild food insecurity (12 months)	0.22	–0.75, 1.42			–0.5	–3.95, 0.55
*Hossain, et al., 2022	Household moderate food insecurity (12 months)	–0.5	–3.95, 0.55			0.51	– 1.53, 0.91
*Hossain, et al., 2022	Household severe food insecurity (12 months)	–0.5	–3.95, 0.55			–7.32	–0.61, –42.05
Food security (long-term follow-up > 1 year)							
**Bossuroy et al., 2022	Food security (18 months)	0.26	0.15, 0.36			0.05	–0.04, 0.15
Haushofer et al., 2020	Food security index (24 months)	0.13	0.01, 0.25	0.23	0.05, 0.41	–0.01	–0.18, 0.16
Needs/Support needs (short-term follow-up <3 months)							
Arean et al., 2005	Needs (post)			0.07	–0.67, 0.81	0.10	–0.56, 0.75
Arean et al., 2005	Support need (post)			–0.08	–0.82, 0.66	–0.31	–0.96, 0.35
Needs/Support needs (medium-term follow-up 3 months to 1 year)							
Arean et al., 2005^3^	Needs (6 months)			–0.46	–1.21, 0.29	–0.24	–0.89, 0.42
Arean et al., 2005	Needs (12 months)			0.11	–0.63, 0.85	–0.14	–0.80, 0.51
Arean et al., 2005^3^	support need (6 months)			0.29	–0.46, 1.03	–0.10	–0.76, 0.55
Arean et al., 2005	support need (12 months)			0.41	–0.34, 1.15	0.15	–0.51, 0.80
Economic performance (short-term follow-up <3 months)							
Blattman et al., (2017, 2022)	Economic performance (2-5 weeks)	1.42	0.96, 1.89	0.94	0.38, 1.50	–0.16	–0.69, 0.38
Economic performance (medium-term follow-up 3 months to 1 year)							
Blattman et al., (2017, 2022)	Economic performance (12-13 months)	0.11	–0.26, 0.49	–0.03	–0.42, 0.36	0.11	–0.43, 0.65
Economic performance (long-term follow-up > 1 year)							
Blattman et al., (2017, 2022)	Economic performance (10 years)	0.39	–0.06, 0.83	0.22	–0.41, 0.85	0.33	–0.30, 0.97
Employment							
Baird et al., 2024	Paid work (12 months)			–0.04	–0.17, 0.09		
Baird et al., 2024	Paid work (24 months)			–0.15	–0.28, –0.02		

* Provided cohen’s d.

** Bossuroy only provided SD of the baseline control group, so that was used for the SD of the dependent variable.

+ Reported results for the marginal effects of poverty-reduction or psychological interventions rather than the effect of each treatment versus control.

3 Excluded from meta-analysis due to repeated assessments within the same follow-up period.

4 Excluded from meta-analysis as multiple conversions using the baseline standard deviation resulted in effect sizes not within the 95% confidence interval.

## Data Availability

All data included in this systematic review are publicly available through the original published studies. A complete list of sources is provided in the references. Extracted data and Hedge’s g calculations are available in the supplementary information.
